# Acute stress disrupts intestinal homeostasis via GDNF‐RET

**DOI:** 10.1111/cpr.12889

**Published:** 2020-08-17

**Authors:** Lin Lin, Bingcheng Feng, Ruchen Zhou, Yi Liu, Lixiang Li, Kairuo Wang, Yanbo Yu, Chao Liu, Xin Long, Xiang Gu, Bing Li, Xiaojie Wang, Xiaoyun Yang, Yingzi Cong, Xiuli Zuo, Yanqing Li

**Affiliations:** ^1^ Department of Gastroenterology Qilu Hospital Cheeloo College of Medicine Shandong University Jinan China; ^2^ Laboratory of Translational Gastroenterology Qilu Hospital Cheeloo College of Medicine Shandong University Jinan China; ^3^ Department of Gastroenterology The Affiliated Hospital of Qingdao University Qingdao China; ^4^ Department of dermatology Peking University People’s Hospital Beijing China; ^5^ Department of Microbiology and Immunology University of Texas Medical Branch Galveston Texas USA

**Keywords:** enterochromaffin cell, intestinal stem cell niche, neurotrophic factor, visceral hypersensitivity

## Abstract

**Objectives:**

Enterochromaffin (EC) cells have been associated with functional gastrointestinal disorders such as IBS. Recently, we found that glial cell‐derived neurotrophic factor (GDNF)‐rearranged during transfection (RET) localized in EC cells in human colonic epithelia. Here, we examine the role of GDNF‐RET in the pathophysiology of diarrhoea‐predominant irritable bowel syndrome (IBS‐D).

**Materials and Methods:**

GDNF was assessed by ELISA and immunohistochemistry in biopsies from IBS‐D patients and healthy controls. Stress was induced by using a wrap‐restraint stress (WRS) procedure to serve as an acute stress‐induced IBS model. The function of GDNF‐RET axis to intestinal stem cell (ISC) homeostasis, and EC cell numbers were assessed in vivo and in vitro.

**Results:**

GDNF‐RET was expressed in EC cells in human colon. GDNF was significantly increased in IBS‐D patients. WRS mice showed increased GDNF‐RET levels in colon. WRS induced visceral hypersensitivity by expanding of ISC and differentiation of EC cell via GDNF‐RET. Furthermore, GDNF‐treated mice recapitulated the phenotype of WRS mice. In vitro, GDNF treatment amplified Wnt signal and increased serotonin levels in colonic organoids in a dose‐dependent manner.

**Conclusions:**

We identified GDNF‐RET was presented in colonic epithelium of patients with IBS‐D. GDNF‐RET played important roles in regulating ISC and EC cell differentiation. Our findings, thus, provide RET inhibitor as new therapeutic targets for treatment of patients with IBS‐D.

## Graphical abstract

GDNF‐RET was expressed in enterochromaffin (EC) cells in human colon. GDNF protein levels were increased in IBS‐D patients. GDNF‐RET indirectly promotes intestinal stem cell (ISC) proliferation through Wnt signal. GDNF‐RET promotes differentiation of ISC, leading to increased density of EC cells in acute stress model.

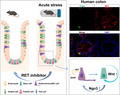

## 1 INTRODUCTION

Irritable bowel syndrome (IBS) is a common functional bowel disorder characterized by abdominal pain with altered bowel habits. The stress has long been considered to play a role in the aetiology of IBS. In most patients, IBS is a chronic relapsing disease which significantly reduces health‐related quality of life. Although its pathogenesis is not completely understood, the role of visceral hypersensitivity in IBS has recently emerged.^1^ 5‐Hydroxytryptamine (5‐HT) released from the enterochromaffin (EC) cells of the gastrointestinal tract is known to play a key role in the physiological states of the gastrointestinal tract.^2^, ^3^ Plasma 5‐HT levels in irritable bowel syndrome with diarrhoea (IBS‐D) patients were higher compared to healthy controls, suggesting a possible role for 5‐HT in the postprandial symptoms.^4^ EC cells are considered to play an important role by acting as chemosensors. It has been shown that EC cells use sensory receptors to detect irritants, metabolites, and catecholamines and convert them into 5‐HT release events.^5^ Rearranged during transfection (RET) has been reported recently to express predominantly in a subset of enteroendocrine cells in mouse intestine.^6^ However, it is still unknown whether intestinal epithelial cells express of RET in human, and the function of RET in EC cells has not yet been identified.

EC cells are terminally differentiated and are unable to replicate themselves.^7^ The Lgr5 + stem cells under intestinal crypts have been shown to long‐term self‐renew and differentiate, giving rise to all cell lineages in the intestinal epithelium.^8^ Mouse atonal homolog 1 (Math1), Neurogenin3 (Neurog3) and NeuroD play prominent roles in ISC fate specification.^9^ Intestinal stem cells (ISC) and progenitors can switch fate choice to increase the proportion of EC cell progenitors, leading to increased EC cell number. However, the mechanisms may be occurring in patients with IBS‐D is unknown.

RET is a transmembrane protein, which binds the members of the glial cell‐derived neurotrophic factor (GDNF) family, and is most commonly implicated in Hirschsprung's disease.^10^ It is well established that RET activity is required for the development of the enteric nerve system,^11^ kidney^12^, ^13^ and spermatogenesis.^14^ Recent studies demonstrate that RET promotes maturation and activates Wnt signalling in the developing mouse intestinal epithelium.^6^ Of interest, in pancreas, GDNF can increase β‐cell proliferation by enhancing Pdx1, Ngn3, NeuroD1/2 and Pax4 gene expression.^15^ These findings suggest that RET may participate in the development of EC cells.

In IBS‐D patients, chronic, low‐grade and subclinical inflammation has been implicated in the disease process.^16^ GDNF has been shown to prevent colonic epithelial cell apoptosis and ameliorate experimental colitis.^17^ In functional dyspepsia, the expression of GDNF is increased in duodenal mucosa.^18^ These results suggested that GDNF might be involved in IBS pathophysiology.

In this report, we showed that colon mucosal GDNF expression was increased in IBS‐D patients, and GDNF‐RET was present in human EC cells. In an acute stress‐induced IBS model, stress induced visceral hypersensitivity by expanding of ISC and differentiation of EC cell via GDNF‐RET, which was inhibited by treatment with RET inhibitor. Moreover, GDNF treatment amplified Wnt signal and increased serotonin levels in colonic organoids in a dose‐dependent manner.

## 2 MATERIALS AND METHODS

### 2.1 Participants and questionnaires

A total of 11 patients with IBS‐D (4 women and 7 men) and 12 control subjects (5 women and 7 men) participated in the study. This study was approved by the Clinical Ethical Committee of the Qilu Hospital of Shandong University, and all participants signed a written informed consent form before participation. The diagnosis of IBS was based on the Rome IV criteria.^19^ Controls were selected from patients undergoing colonoscopy for cancer surveillance who received negative results. Exclusion criteria were as follows: patients taking non‐steroidal anti‐inflammatory drugs or other anti‐inflammatory drugs (including mast cell stabilizers, histamine antagonists, probiotics, immunosuppressants and steroids); had undergone major abdominal surgery; or had any organic syndrome, including coeliac disease, allergic diseases and psychiatric disorders as assessed by history taking, appropriate consultations and laboratory tests. All specimens were taken from the rectosigmoid junction to standardize the site of sampling.

### 2.2 Organoid cultures

The small intestines and colons of male C57BL/6 mice were isolated and cut into 2‐4 mm sections after making a longitudinal incision along the entire length of the intestine, then incubated in 2 mmol/L ethylenediaminetetraacetic acid (EDTA) on a rocking platform at 100 rpm for 30 minutes (small intestine) or 60 minutes (colon) at 4°C. The tissue was then re‐suspended in clean PBS and pipetted several times. The supernatant was collected and filtered through a 70 μm filter, then centrifuged at 300 × *g* for 5 minutes at 4°C. Isolated crypts were suspended in Matrigel Matrix (Corning, NY, USA). The suspension was carefully pipetted into the centre of each well in a pre‐warmed 24‐well plate (Corning). After the Matrigel solidified, IntestiCult^™^ Organoid Growth Medium (STEMCELL Technologies, Vancouver, Canada) supplemented with penicillin‐streptomycin (100 units/100 μg per mL) was added and refreshed every 3‐4 days. Organoids were kept in a humidified atmosphere containing 5% CO_2_ at 37°C.

Organoid treatment: organoids cultured for 2 days were incubated with recombinant murine GDNF (PeproTech, London, UK) at different concentrations (50, 100 and 200 nmol/L) or RET kinase inhibitor GSK3179106 (MedChemExpress, La Jolla, CA) at 100 nmol/L for 96 hours. After stimulation, supernatants were used for the following analysis, and organoids were lysed for RNA isolation.

### 2.3 Animal models

Adult male C57BL/10 mice were purchased from the animal centre of Shandong University of Traditional Chinese Medicine. All experiments were approved by the Ethical Committee and Institutional Animal Care and Use Committee of Qilu Hospital. For the acute stress model, stress was induced by using a wrap‐restraint stress (WRS) procedure, an acute non‐ulcerogenic model of colonic hypersensitivity. All constraint was performed at the same time of the day, between 10 am and 12 am for 7 days, to minimize the influence of circadian rhythms. We forced immobilization of mice by placing them in 50 mL tubes with a hole for air. Mice were then placed back in their home cage.

For oral dosing, GSK3179106 was prepared as a suspension in saline at 1 μg/mL and administered at 10 μg/kg and injected BID at 8 am and 4 pm for 2 days. For intraperitoneal injection, recombinant murine GDNF was administrated at 10 μg/kg and injected BID for 5 days. After 7 days of administration and injection, the mice were evaluated by colorectal distension (CRD) test.

### 2.4 Visceral hypersensitivity evaluation

The visceral hypersensitivity was evaluated by CRD test, following the previously described protocol.^20^ The distension was applied on awake mice by using a 4 mm Fogarty catheter balloon inserted into the descending colon. Graded CRD was performed by rapidly injecting different volumes of normal saline (0, 0.04 0.06, 0.08, 0.1, 0.12, 0.14, 0.16, 0.18. 0.2 and 0.22 mL) into the balloon and maintaining the distension for 20 seconds. Distension was given at 4 minutes intervals and repeated 3 times to achieve an accurate result. The abdominal withdrawal reflex (AWR) score was recorded according to a method described before,^21^ and the mean scores from three data were used as the final score.

### 2.5 Immunohistochemistry

Endoscopic biopsy colon tissues and mice colon were fixed overnight at 4°C in 4% paraformaldehyde and was subsequently embedded in paraffin. Fixed and embedded tissues were sectioned (4 μm) into slides. Immunohistochemical staining of human colon biopsy was performed using rabbit anti‐GDNF (1:500, Abcam, Cambridge, UK), rabbit anti‐ChgA antibody (1:300; Abcam), rat anti‐serotonin antibody (1:500; Abcam) and mouse anti‐RET antibody (1:300; Abcam). Mice colon sections were performed using rat anti‐serotonin antibody (1:500; Abcam).

### 2.6 Enzyme‐linked immunosorbent assays

The total proteins of biopsy colon tissues were extracted and centrifuged, and the supernatants were taken out and quantified. The protein level of GDNF was measured using human GDNF ELISA kit (Abcam) according to the manufacturer's instructions. After organoid treatment, the medium was taken out and centrifuged to furnish the supernatant. The protein level of 5‐HT in the medium was measured using Serotonin ELISA kit (Abcam) according to the manufacturer's instructions. Samples and standards were added in duplicate to 96‐well ELISA plates.

### 2.7 Immunofluorescence staining

The slides were incubated overnight at 4°C with the primary antibody, washed in PBS and incubated with secondary antibodies for 1.5 hours at room temperature. This was followed by washing with PBS and mounting with Vectashield‐DAPI mounting medium. Primary antibodies used in our experiment were as follows: mouse anti‐RET antibody (1:100; Santa Cruz, Santa Cruz, CA) and rabbit anti‐ChgA antibody (1:200; Abcam). Secondary antibodies were Alexa Fluor 647‐conjugated anti‐mouse IgG (1:400; Abcam) and Alexa Fluor 488‐conjugated anti‐rabbit IgG (1:400; Abcam), and DAPI (Abcam). Images were taken on Zeiss LSM 880 with an AiryScan confocal laser scanning microscope and were analysed with zen 2009 Light Edition software.

### 2.8 qPCR

Total RNA was extracted from organoid using the RNAprep Pure Cell/Bacteria Kit (TIANGEN, Beijing, China) according to the manufacturer's protocol. Similarly, total RNA of tissue specimens was extracted using RNAprep pure Tissue Kit (TIANGEN) and cDNA was synthesized using ReverTra Ace qPCR RT Kit (Toyobo, Osaka, Japan). All reactions were run in triplicate on an Applied Biosystems StepOne Real‐Time PCR System (Thermo, Waltham, MA, USA). The primer sequences used for real‐time PCR analysis are listed in Table 1.

**Table 1:** Primer sequences for qPCR

Gene	Primer forward	Primer reverse
*Sox9*	CTGGAGGCTGCTGAACGAGAG	CGGCGGACCCTGAGATTGC
*Cd44*	TCGATTTGAATGTAACCTGCCG	CAGTCCGGGAGATACTGTAGC
*Axin2*	GAGAGTGAGCGGCAGAGC	CGGCTGACTCGTTCTCCT
*Lgr5*	CGAGCCTTACAGAGCCTGATACC	TTGCCGTCGTCTTTATTCCATTGG
*Gdnf*	AAAGACTGAAAAGGTCACCAGA	CAAACCCAAGTCAGTGACATTT
*Tph1*	TCCGTCCTGTGGCTGGTTACC	AGGTGTCTGGCTCTGGAGTGTAG
*Ngn3*	TTTGAGTCGGGAGAACTAGGATGG	TTGGAACTGAGCACTTCGTGGT
*NeuroD*	AGGAATTCGCCCACGCAGAA	TGGTCATGTTTCCACTTCCTGTTGT
*β‐actin*	TCTGTGTGGATTGGTGGCTCTA	CTGCTTGCTGATCCACATCTG

### 2.9 Western blot

Total proteins were extracted and quantified. Protein was separated by 10% SDS‐PAGE and transferred onto a PVDF membrane. The membrane was incubated with primary antibody at 4°C overnight, then incubated with the secondary antibody for 1 hour at room temperature. The immunoblots were detected by an enhanced chemiluminescent substrate (Millipore). Antibodies: anti‐RET antibody (1:1000; Abcam), anti‐Lgr5 antibody (1:1000; Abcam), anti‐β‐catenin antibody (1:1000; Proteintech, USA), anti‐TPH1 antibody (1:500; Abcam), anti‐GDNF antibody (1:500; Abcam), anti‐Ngn3 antibody (1:300; Abcam), anti‐β‐actin antibody (1:1000; Zhongshan Gold Bridge, Beijing, China), goat anti‐mouse IgG (1:1000; Zhongshan Gold Bridge) and goat anti‐rabbit IgG (1:1000; Zhongshan Gold Bridge).

### 2.10 Statistical analysis

Branching coefficient was assessed according to previous described formula^6^:

$$\text{Branching}\;\text{coefficient}=1‐\text{circularity}=1‐\frac{4\pi \;\text{area}\;}{{\text{primeter}}^{2}}.$$

Results were analysed using graphpad prism 5.0c (GraphPad Software, La Jolla, CA, USA). Univariate analysis of the characteristics of the population used the chi‐square test. Differences between multiple groups were evaluated using one‐way ANOVA. Student's *t* test or Mann‐Whitney non‐parametric test was applied to compare two groups. Correlations between the two parameters were assessed by Spearman rank correlation. Results were expressed as means ± SD. Differences were considered significant at *P* < .05.

## 3 RESULTS

### 3.1 Demographics and clinical characteristics

Demographics and clinical characteristics of control subjects and patients with IBS‐D are shown in Table 2. There was no significant difference in age, sex, or the body mass index (BMI) between the control group and IBS‐D group. In IBS‐D group, the anxiety subscale scores (control vs IBS‐D: 2.3 ± 0.8 vs 4.8 ± 1.7, *P* < .05) and depression subscale scores (control vs IBS‐D: 2.6 ± 1.2 vs 4.1 ± 1.8, *P* < .05) were significantly higher.

**Table 2:** Demographic and clinical characteristics of study subjects

	Controls (n = 12)	IBS‐D patients (n = 11)	*P* value
Age (y)	43.7 ± 11.3	47.7 ± 12.8	.428
Sex, M/F	7/5	7/4	1.000
BMI, kg/m^2^	25.4 ± 2.4	24.6 ± 2.8	.468
IBS duration (y)	‐	4.2 ± 2.6	‐
IBS‐SSS	‐	229.2 ± 60.9	‐
HADS anxiety scores	2.3 ± 0.8	4.8 ± 1.7	**.001**
HADS depression scores	2.6 ± 1.2	4.1 ± 1.8	**.026**

Note

Quantitative data are expressed as mean ± SD.

Bold value is statistically significant *P* < .05.

Abbreviations: BMI, body mass index; F, female; HADS, hospital anxiety and depression scale; IBS‐D, irritable bowel syndrome with diarrhoea; IBS‐SSS, irritable bowel syndrome severity scoring system; M, male.

### 3.2 Human colonic epithelial cells express GDNF‐RET

**Figure 1:**
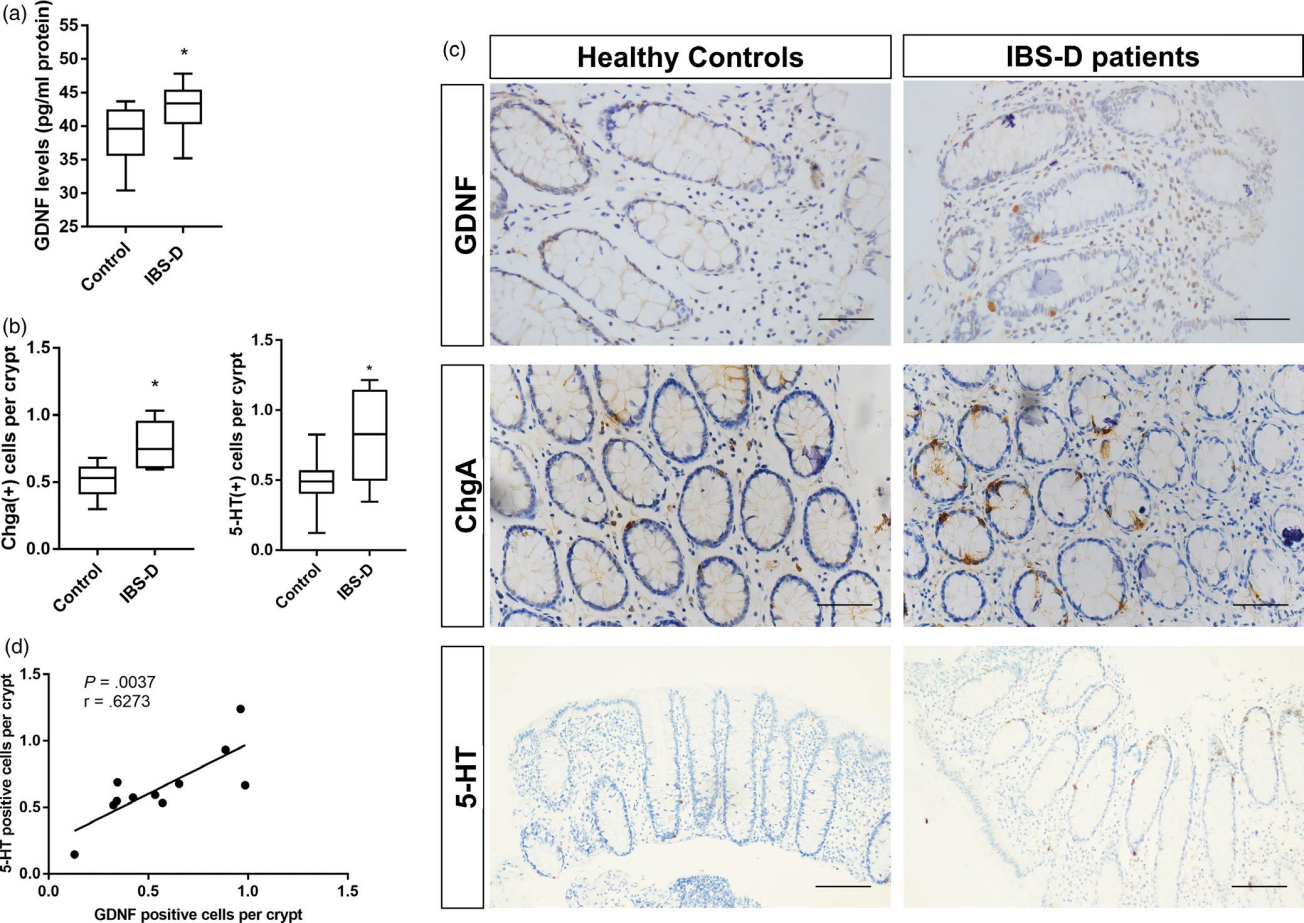
IBS‐D patients showed reduced GDNF protein levels. A, Expression of GDNF in colonic biopsies of patients with irritable bowel syndrome with diarrhoea (IBS‐D) (n = 11) and control subjects (n = 12). B, Quantification of ChgA‐positive and serotonin‐positive cells in colonic biopsies from patients with IBS‐D and controls. C, Representative photomicrographs showing positive GDNF, Chga and 5‐HT immunoreactivity in the colonic mucosa of a control and a patient with IBS‐D. Scale bar: 50 μm and 100 μm (5‐HT). D, Correlation between the number of 5‐HT‐positive cells and GDNF‐positive cells in the human colonic mucosa. The data were displayed as mean ± SD; *P < .05

**Figure 2:**
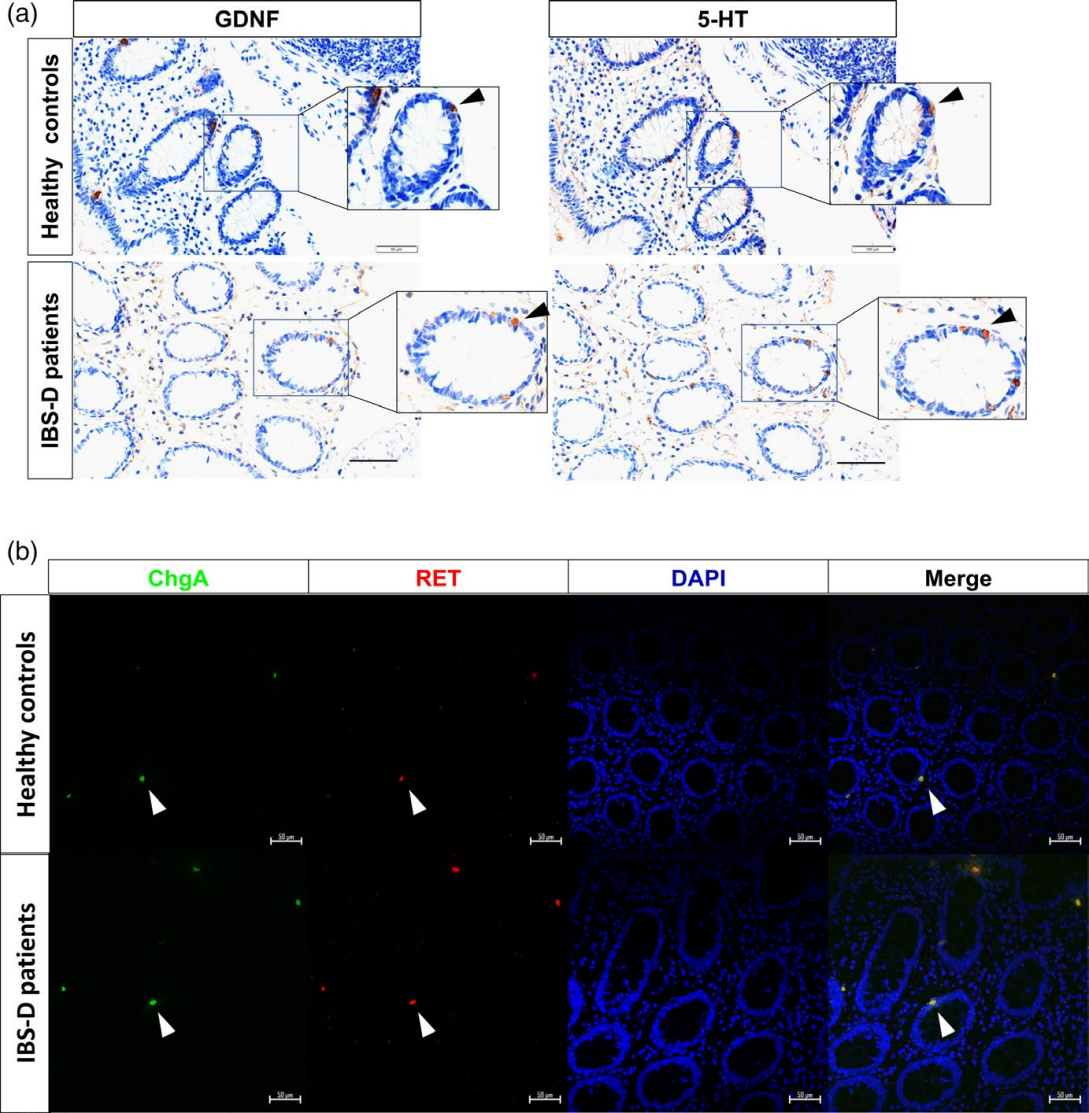
Localization of GDNF‐RET in EC cells. A, Representative photomicrographs showing positive GDNF and 5‐HT immunoreactivity in two serial sections of biopsy tissues in both IBS‐D patients and healthy controls. Scale bar: 50 μm. B, Double‐label immunofluorescence analysis of ChgA (green) and Ret (red) in lamina propria of human colon in both IBS‐D patients and healthy controls. Same field showing DAPI (blue). Merged image showing co‐localization (yellow) of ChgA and RET immunoreactivities. Scale bar: 50 μm

GDNF protein levels in endoscopic biopsy specimens from patients with IBS‐D and healthy controls were compared quantitatively by ELISA. In IBS‐D patients, GDNF protein was significantly increased in the colonic mucosa compared with controls (38.78 ± 1.25 vs 42.60 ± 1.22 pg/mg protein, respectively) (Figure 1A). Since enteric glial cells have long been considered as the main source for secreting GDNF, we determined the localization of GDNF expression in the intestines. Interestingly, immunohistochemistry showed that GDNF was not only localized in interstitial cells but also enriched in colonic epithelial cells that scatter among colonic mucosa (Figure 1B). Furthermore, we evaluated alterations of colonic ChgA expression, which is a marker for enteroendocrine cell, in control and IBS‐D patient group. Consistent with previous studies, the numbers of ChgA‐expressing and 5‐HT‐expressing epithelial cells of IBS‐D patients were both significantly increased (Figure 1C). Notably, GDNF‐positive cells were similar in shape to ChgA‐positive or 5‐HT‐positive cells, with a narrow apex reaching to the lumen and a wider base. Furthermore, the number of GDNF‐producing epithelial cells and the number of serotonin‐positive epithelial cells were positively correlated (Figure 1B,D). We then investigated whether GDNF is gathered and packed in granules of enterochromaffin cells. Firstly, two serial sections of biopsy tissues were incubated with anti‐GDNF antibody and anti‐serotonin antibody, respectively. As expected, GDNF was mainly concentrated in EC cells in epithelium (Figure 2A). Next, we localized GDNF receptor RET‐expressing cells in human colonic mucosa. Immunofluorescence showed that RET‐positive epithelial cells corresponded to a subset of ChgA‐positive cells (Figure 2B), indicating that RET‐positive cells were predominantly EC cells in human colonic mucosa, which is consistent with the results in a recent study in mice.^6^ Put all together, these data suggested that GDNF‐RET is expressed in EC cells of the human colonic mucosa and possibly participates in the hyperplasia of EC cells.

### 3.3 Acute adult stress induces proliferation of EC cells in mice colon

**Figure 3:**
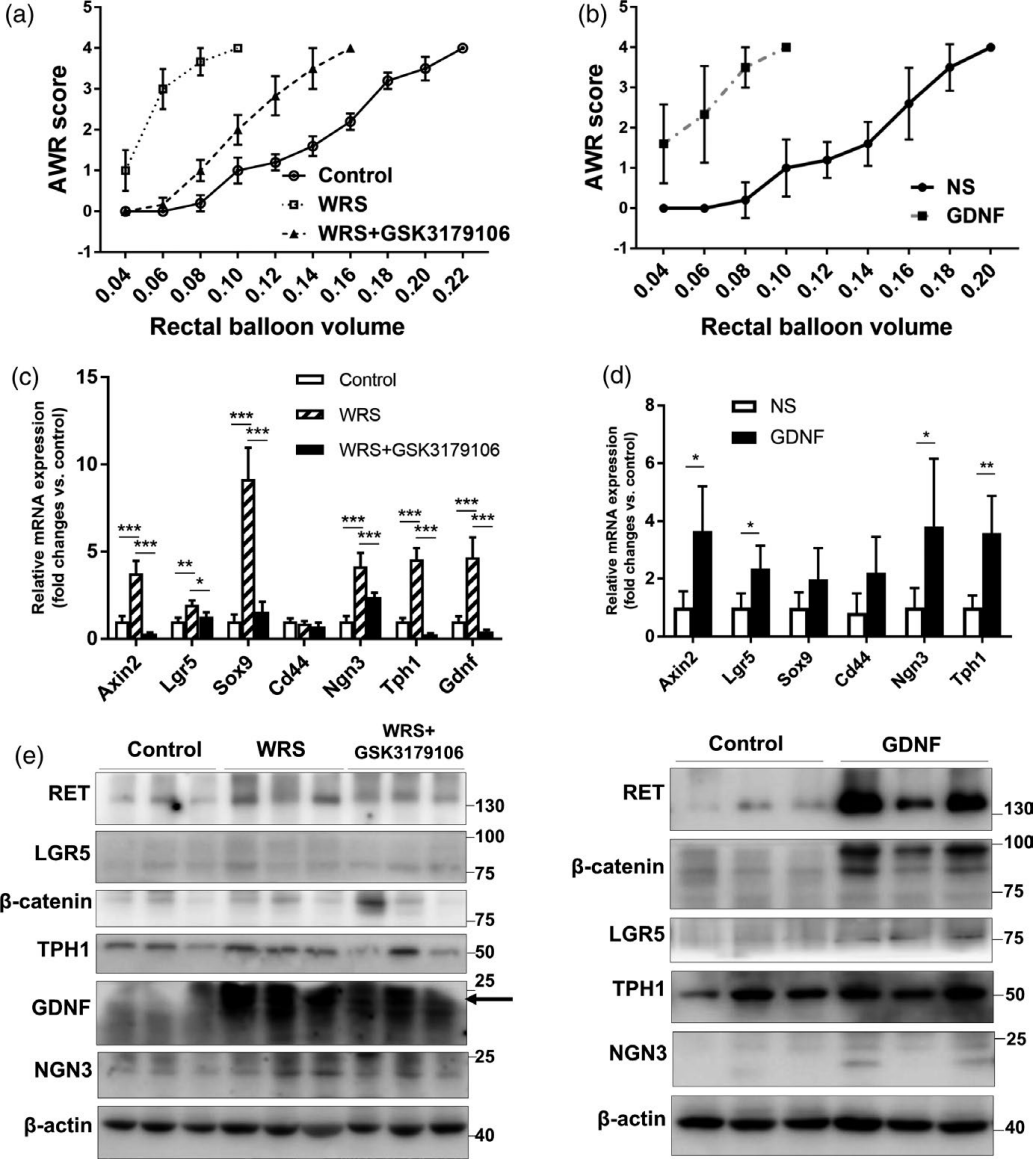
WRS promoted ISC expansion mediated by GDNF‐RET. A, Abdominal withdrawal reflex (AWR) scores in response to CRD at different pressures; control (n = 5), WRS (n = 9) and WRS with GSK3179106 gavage (n = 7). B, AWR scores in response to CRD at different pressures between GDNF intraperitoneal injection group (n = 5) and saline intraperitoneal injection group (n = 6). C, The relative expression of Axin2, Lgr5, Sox9, Cd44, Tph1 and Gdnf mRNA levels in different groups, reported as fold changes of control group. D, The relative expression of Axin2, Lgr5, Sox9, Cd44, Tph1 and Ngn3 mRNA levels in GDNF intraperitoneal injection group and saline intraperitoneal injection group, reported as fold changes of control group. E, Representative pictures of RET, LGR5, β‐catenin, TPH1, GDNF, NGN3 and β‐actin expression in different groups. GDNF target signal is tagged with black arrow. The data were displayed as mean ± SD; *P < .05, **P < .01 and ***P < .001

**Figure 4:**
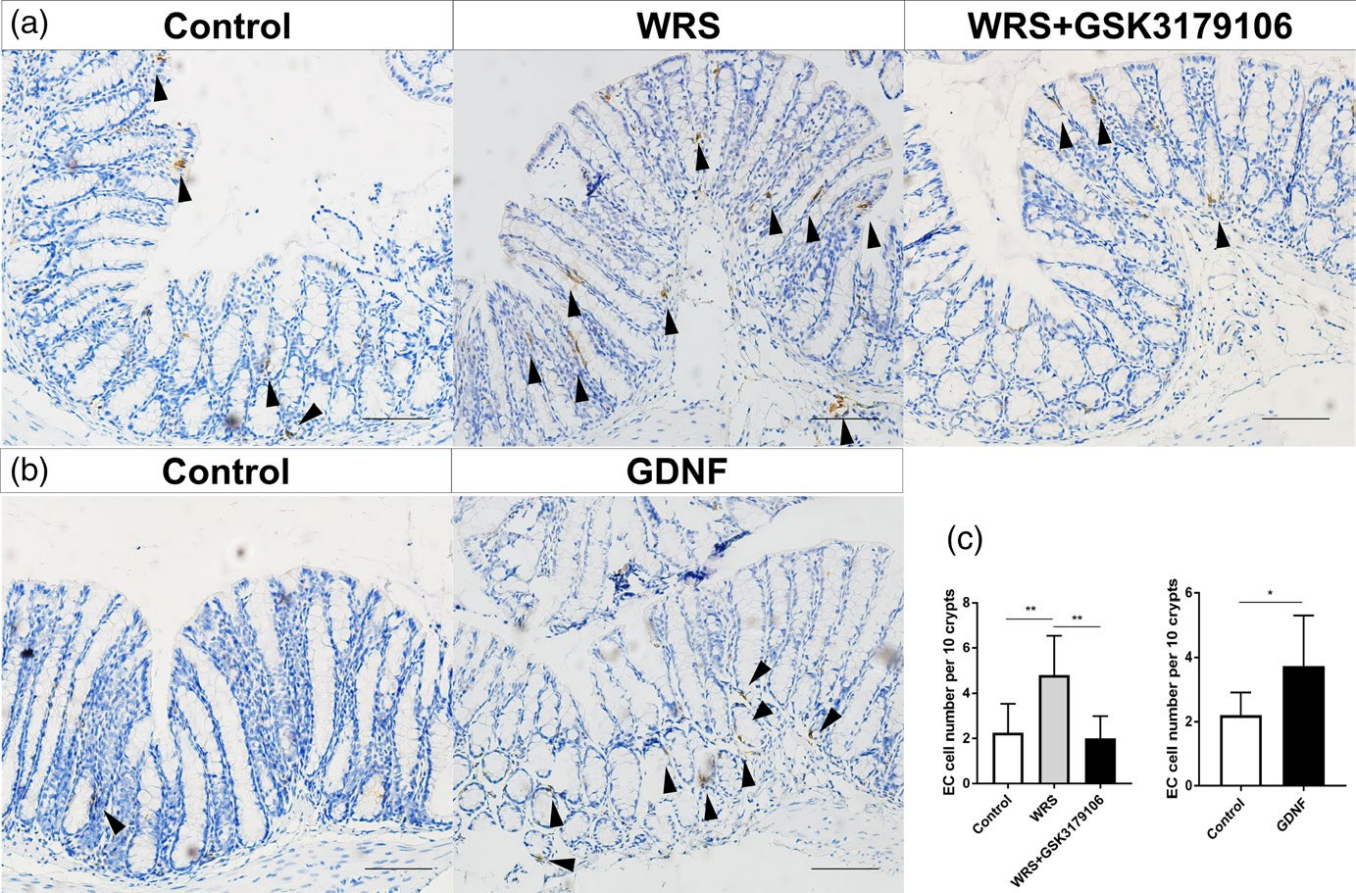
WRS promoted EC expansion. A, Representative photomicrographs showing positive 5‐HT immunoreactivity (black arrowhead) in the colonic mucosa of control mice, WRS mice and WRS mice with GSK3179106 gavage. Scale bar: 100 μm. B, Representative photomicrographs showing positive 5‐HT immunoreactivity (black arrowhead) in the colonic mucosa of saline intraperitoneal injection mice and GDNF intraperitoneal injection mice. Scale bar: 100 μm. C, WRS/GDNF‐treated mice showed increased number of 5‐HT‐positive cell in colonic mucosa. GSK3179106 oral gavage significantly decreased the number of 5‐HT‐positive cell to baseline. The data were displayed as mean ± SD; *P < .05 and ** *P* < .01

To investigate the potential role of GDNF in acute stress‐related disorders in the intestine, we adopted a WRS model which was an adequate model to mimic part of the main symptoms of IBS, such as pain and colonic dysmotility. In WRS mice, a statistically significant increase in the AWR score was shown in volume 0.06 mL and 0.08 mL, *P* < .001. WRS treatment generated a sharper curve compared to control, and reached score 4 at small balloon volume, indicating a higher colorectal sensitivity (Figure 3A). WRS significantly increased the expression of GDNF in the colon. The expression of RET in the colon was also upregulated by WRS (Figure 3E). To investigate whether GDNF could induce colorectal hypersensitivity, we treated mice with recombinant GDNF intraperitoneally. Compared with saline controls, recombinant GDNF treatment caused higher visceral sensitivity (Figure 3B). As hyperplasia of EC cells was found in IBS patients, we examined the density of EC cells in both WRS mice and GDNF injection mice. Interestingly, both WRS and GDNF stimulation increased EC cell expansion in the colon (Figure 4A‐C). Furthermore, the expression of TPH1, the rate‐limiting biosynthesis enzyme of 5‐HT in the EC cell, was upregulated by the WRS and GDNF treatment (Figure 3C‐E). In addition binding to RET, GDNF also binds two other receptors, NCAM^22^ and syndecan‐3.^23^ To determine whether GDNF promotion of the EC cell expansion is mediated specifically by RET, WRS mice were treated with GSK3179106, a gut‐restricted RET kinase inhibitor,^24^ by oral gavage. Blocking GDNF‐RET in WRS mice effectively reduced the visceral nociception (Figure 3A). Moreover, inhibiting RET not only reduced serotonin‐positive cells in the colon but also reduced the expression of TPH1 and GDNF (Figure 3C,E). Put together, these results indicated that acute adulthood stress resulted in EC cell expansion via GDNF‐RET axis.

### 3.4 GDNF‐RET promotes intestinal stem cell proliferation and differentiation during acute adulthood stress

**Figure 5:**
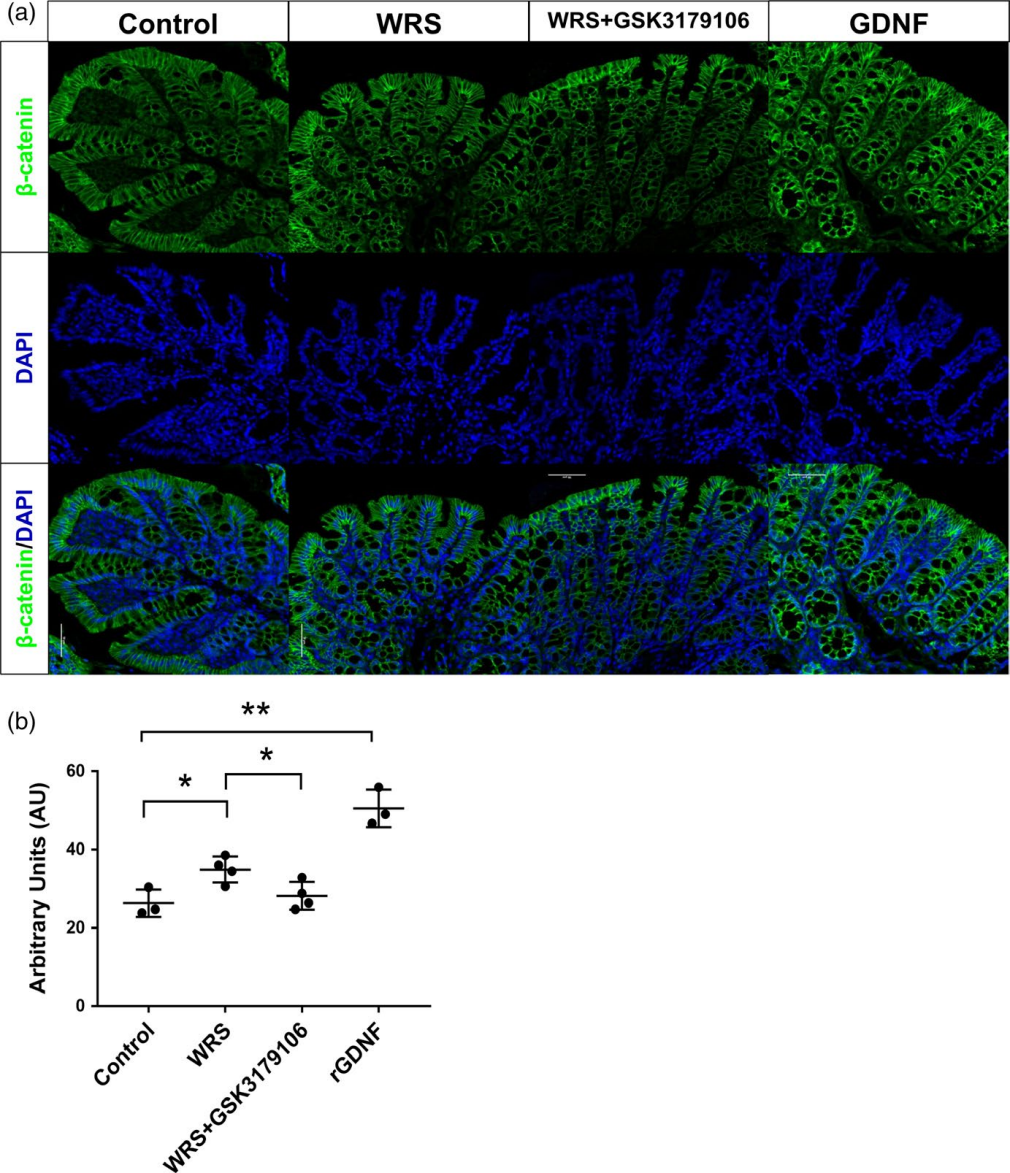
WRS promoted Wnt signalling. A, Immunofluorescence of β‐catenin (green) in the sections of the proximal colon from control mice, WRS mice treated with or without GSK3179106 and mice with GDNF intraperitoneal injection. Scale bars: 100 μm. B, The quantification of fluorescence intensity in indicated groups

RET has been previously reported to promote maturation and Wnt signalling in developing mouse intestinal epithelium.^6^ We speculated that GDNF‐RET enhanced EC cell expansion by inducing proliferation of intestinal stem cells. We first examined the expression of Lgr5, an intestinal stem cell marker. Both WRS and GDNF stimulation significantly promoted Lgr5 expression in mRNA and protein levels. Additionally, inhibition of RET in WRS mice effectively suppressed Lgr5 expression (Figure 3C‐E). RET could amplify Wnt signal in the intestine epithelium, promoting us to confirm in WRS/GDNF‐treated mice. The expression of Axin2 and β‐catenin, which have been involved in the cascade events of Wnt signal activation, was increased in WRS/GDNF treatment group (Figure 3C‐E). Additionally, immunostaining revealed that the expression of β‐catenin was increased in the colon mucosa from WRS‐ and GDNF‐treated mice. Inhibiting GDNF/RET by GSK3179106 suppressed the expression of β‐catenin (Figure 5). Sox9 plays an important role in regulating cell proliferation and modulating Wnt pathway by acting in a feedback loop.^25^, ^26^ CD44, one of the target genes for Wnt/β‐catenin pathway,^27^ is thought to function as a cell adhesion receptor linking hyaluronate to the cell and the cytoskeleton. The expression of Sox9 and CD44 was both upregulated by WRS/GDNF treatment (Figure 3C‐E). Furthermore, GSK3179106 administration reduced the expression levels of Axin2, β‐catenin, Sox9 and CD44 (Figure 3C‐E). Put all together, these data indicated that GDNF amplifies Wnt/β‐catenin signal via RET in the colon epithelium.

GDNF influences β‐cell proliferation by enhancing Ngn3 expression in the pancreas,^15^ and Ngn3 is required for EC cell fate specification in intestine.^28^ We then investigated whether GDNF/RET axis also regulates EC cell differentiation in the intestine. As shown in Figure 3C‐E, the expression of Ngn3 was increased in WRS‐ and GDNF‐treated mice. The Ngn3 expression was inhibited by the treatment with GSK3179106, suggesting that GDNF‐induced stem cell differentiation was mainly mediated by RET (Figure 3C‐E).

### 3.5 GDNF‐RET promotes maturation of ISC

**Figure 6:**
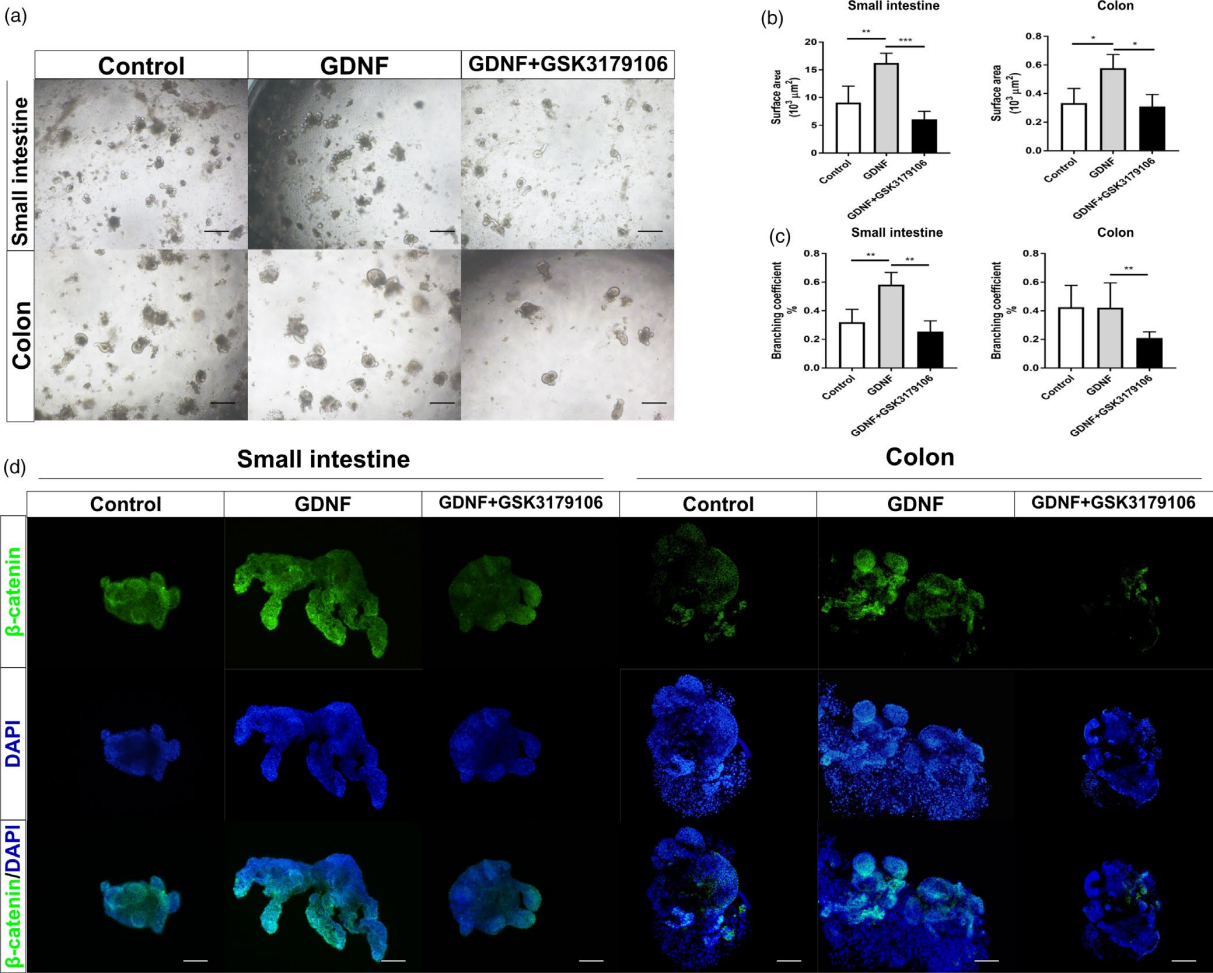
GDNF‐RET induced ISC self‐renew in colonic organoids. A, Representative images of small intestinal and colonic organoids stimulated by GDNF and GDNF with GSK3179106 pre‐incubation. B, Surface area of organoids in (A). C, Branching quantifications of organoids in (A). D, Immunofluorescence of β‐catenin (green) in small intestinal and colonic organoids stimulated by GDNF and GDNF with GSK3179106 pre‐incubation. The data were displayed as mean ± SD; *P < .05, **P < .01 and ***P < .001

**Figure 7:**
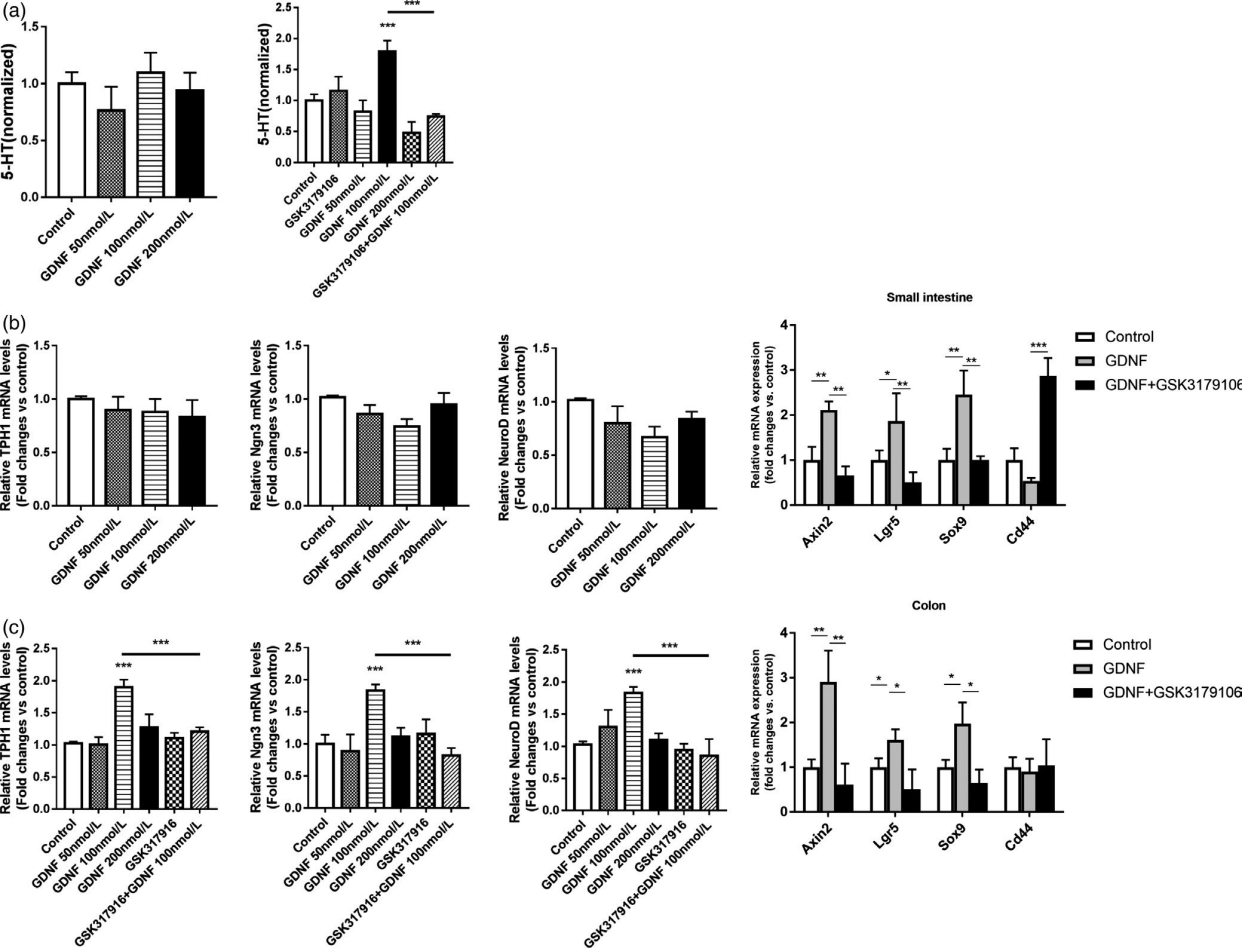
GDNF‐RET regulated epithelial maturation in colonic organoids. A, Effects of GDNF and RET inhibitor GSK3179106 on the 5‐HT secretion in small intestinal and colonic organoids. GDNF concentrations were 50, 100 and 200 nmol/L. GSK3179106 were pre‐incubated 1 h before GDNF stimulation. The 5‐HT levels in intestinal organoids supernatant. Data were normalized to 5‐HT levels in Control group. B, The Tph1, Ngn3, NeuroD, Axin2, Lgr5, Sox9 and Cd44 mRNA expression in small intestinal organoids. C, The Tph1, Ngn3, NeuroD, Axin2, Lgr5, Sox9 and Cd44 mRNA expression in colonic organoids. The data were displayed as mean ± SD; *P < .05, **P < .01 and ***P < .001

In vitro, organoids from small intestine and colon were exposed to GDNF and RET inhibitor. The increased proliferation of ISC induced by GDNF/RET was evidenced by the increased branching efficiency and the size in GDNF‐treated organoids, and their reduced branching and size in GSK3179106‐treated organoids (Figure 6A‐C). We further examined the distribution of β‐catenin by immunofluorescence, GDNF‐treated organoids have increased levels of β‐catenin, while GSK3179106 prevented the increase in both small intestinal and colonic organoids (Figure 6D). Intriguingly, treatment with GSK3179106 decreased the numbers of buds and was more likely for organoids to forming a sphere. Since GDNF promotes the specification of EC cell in vivo, we measured the 5‐HT in supernatants of organoids stimulated with different concentrations of GDNF. The increase in 5‐HT was observed in 100 nmol/L GDNF‐treated colonic organoids and reduced by pre‐incubation of GSK3179106. The secretion of serotonin by intestinal organoids did not respond to changes of GDNF (Figure 7A). Notably, 200 nmol/L GDNF significantly suppressed the secretion of 5‐HT in colonic organoids, indicating a bidirectional function of GDNF. Consistently, mRNA level of THP1 was increased in GDNF‐treated colonic organoids but not in GDNF‐treated small intestinal organoids (Figure 7B,C). Transcriptional analysis revealed an increase in Lgr5 and Sox9, two ISC markers, in colonic organoids stimulated with 100 nmol/L GDNF. Moreover, the expression of Wnt‐related genes, such as Axin2 and Cd44, were also upregulated in 100 nmol/L GDNF‐treated colonic organoids (Figure 7B,C). Pre‐incubation of GSK3179106 significantly reduced the expression of Wnt‐related genes to the levels similar to controls (Figure 7B,C). To confirm the role of GDNF/RET in regulating EC cell specification, we examined the expression of Ngn3 and NeuroD, two genes critical in the specification and differentiation of enteroendocrine cells. GDNF upregulated the expression of Ngn3 and NeuroD at 100 nmol/L (Figure 7B,C). These data implied that GDNF/RET upregulates proliferation and differentiation of ISC in a dose‐dependent manner.

## 4 DISCUSSION

Epithelial barrier defects are observed in IBS‐D patients with associated tight junction alterations.^29^ GDNF affects the apical junction complex, resulting in the maturation of epithelial barrier function by autocrine and paracrine regulatory mechanisms.^30^ GDNF also has a strong anti‐apoptotic effect on colonic epithelial cells and is increased in mucosa of inflammatory bowel disease (IBD) patients.^31^ In this study, we demonstrated that GDNF expression was increased in IBS‐D patients. Although the enteric glial cells (EGC) are well established as the major resource for GDNF production,^32^ unexpectedly, we observed the amplification of GDNF in specific epithelial cells similar in shape to the enterochromaffin cells of the human colonic mucosa. Moreover, the number of GDNF‐positive cells was positively correlated with the number of serotonin‐positive cells. Furthermore, a co‐localization of GDNF and RET in EC cells in human colon was also observed. Considering that EC cells contribute 90% of the serotonin production in circulation and regulate the gastrointestinal motility and secretions, the increased GDNF‐RET may participate in the pathogenesis of IBS‐D by regulating EC cells. Recently, a real‐time single‐cell sequencing survey showed that RET changed significantly in the intermediate and late stages of enteroendocrine differentiation.^33^ However, how GDNF is produced, packaged and transported to EC cells are worth further investigation.

Stress has long been considered as an important factor for the onset and exacerbation of IBS, which is manifested as psychiatric disorders such as anxiety and depression and pain. In our study, elevated GDNF protein levels were shown in WRS model mice, an acute stress induced EC cell hyperplasia in adulthood, resulting from ISC proliferation and differentiation to EC cell. Given that GDNF treatment led to the changes in the ISC compartment similar to that in WRS mice, it appears that GDNF accelerates the self‐renewal of the ISC.

To explore the function of RET on EC cells, a potent, selective and gut‐restricted small molecule RET kinase inhibitor, GSK3179106, was applied in mice and organoids. It has been reported that this inhibitor can attenuate colonic contraction by maintaining cholinergic neuronal function in rats^34^ and works as a clinical candidate for the treatment of IBS.^24^ However, the effect of RET inhibitor on intestinal epithelial cells has not been illustrated. Given that GSK3179106 effectively ameliorated stress‐induced EC cell expansion, the GDNF‐RET axis seemed to be a potential therapeutic target for acute stress‐induced hypersensitivity.

Our current study showed that GDNF increased 5‐HT levels in supernatants of colonic organoids in a dose‐dependent way, which was inhibited by addition of GSK3179106. A previous study showed that GDNF intracerebroventricular injection could upregulate the expression of tryptophan hydroxylase‐2 (TPH2) in the midbrain of antidepressants sensitive cataleptics mice.^35^ In line of this finding, we examined the expression of TPH1 in colonic organoids. GDNF induced an increase in TPH1 mRNA level whereas GSK3179106 pre‐incubation abrogated such effect, indicating that activation of the 5‐HT system was mediated by RET kinase. Interestingly, the GDNF treatment did not affect the activation of 5‐HT and TPH1 expression in small intestinal organoids. This regional specificity has also been shown with microbiota‐induced 5‐HT biosynthesis, which occurs in colonic, but not small intestinal EC cells.^36^ As the differential expression of specific nutrient transporters and receptors in small intestinal and colonic EC cells,^37^ the ability of EC cells in sensing stimulation may vary along the GI tract.

Wnt signalling has recently been found to be crucial for proliferation, differentiation, and migration of the stem cells^38^ and is crucial in intestinal organoids culture.^39^, ^40^ In APC^min^ mice where Wnt signalling is constitutively active, the number of EC cells in colon is significantly increased.^41^ A recent study proposes that there is positive feedback between RET and Wnt signalling in *Drosophila* and GDNF upregulated Axin2 expression in mice colonic organoids.^6^ Consistently, we showed that GDNF‐RET amplified Wnt signalling and increased ISC expansion. Furthermore, GDNF‐RET likely promoted ISC differentiation to enteroendocrine cell line by regulating Ngn3 and NeuroD. All together, these data suggest that GDNF‐RET may participate in expansion of colonic ISC via Wnt signalling.

In conclusion, our study demonstrated the roles of GDNF‐RET axis in regulation of ISC self‐renewal state and differentiation, which plays an important role in the pathophysiology of IBS‐D. EC cell might be a potential cell for ISC niche. Further investigation into the role of RET in visceral hypersensitivity may provide a novel potential therapeutic target for treatment of patients with IBS.

## CONFLICT OF INTEREST

All authors declare no conflicts of interests.

## AUTHOR CONTRIBUTIONS

LL and LXL designed experiments; LL, BCF and RCZ performed the experiments, collected data and interpreted data; LL wrote manuscript; KRW, BL, XL and XG participated in acquisition of data and analysis of data; CYZ, YBY and XW revised the manuscript; CL, XYY and YL diagnosed the patients and provided clinical information and samples. YQL and XLZ designed study, obtained funding, revised of the manuscript and approved the final version to be published.

## Data Availability

The data that support the findings of this study are available from the corresponding author upon reasonable request.
